# Supplementary material for: Acceptance rate and risk perception towards the COVID-19 vaccine in Botswana
Source: PLoS One. 2022 Feb 4;17(2):e0263375. doi: 10.1371/journal.pone.0263375 (PMC8815939; doi:10.1371/journal.pone.0263375)
Supplement: S1 Questionnaire — (PDF) [file pone.0263375.s002.pdf]

Republic of Botswana
Ministry of Health and Wellness

# COVID-19 QUESTIONNAIRE
# ACCEPTANCE AND RISK PERCEPTIONS TOWARDS THE COVID-19 VACCINE IN BOTSWANA

## SECTION A – DEMOGRAPHIC INFORMATION

**A1. Nationality**

- [ ] Motswana
- [ ] Other: ____________________

**A2. How old are you at your last birthday?** ____________________

**A3. Sex**

- [ ] Male
- [ ] Female
- [ ] Other

**A4. Marital Status**

- [ ] Single
- [ ] Married
- [ ] Divorced
- [ ] Widowed

**A5. Residence**

- [ ] Rural
- [ ] Semi-Urban
- [ ] Urban

**A6. Occupation**

- [ ] Employed (Government, Private, Business/Self)
- [ ] Unemployed
- [ ] Student
- [ ] Others

*Turn Page Over*

**A7. Educational Level**

- [ ] Primary
- [ ] Junior Secondary
- [ ] BGCSE/ IGCSE
- [ ] Under Graduate
- [ ] Post Graduate

**A8. Religion**

- [ ] Christian
- [ ] Hindu
- [ ] Islam
- [ ] Buddhism
- [ ] Other ____________________

**SECTION B – COGNITIVE FACTORS**

**B1. Are you suffering from any of the following? (tick more than 1 if applicable)**

- [ ] Hypertension
- [ ] Diabetes Mellitus
- [ ] Cancer
- [ ] Asthma
- [ ] Cardiovascular disease
- [ ] Chronic respiratory
- [ ] Other ____________________
- [ ] None

**B2. Are you aware of the covid-19 vaccine?**

- [ ] Yes
- [ ] No

**B3. Would the vaccine alone protect you against COVID-19 disease?**

- [ ] Yes
- [ ] No

*Turn Page Over*

**B4. The vaccine is recommended for those 18 years and above only in Botswana**

- ☐ True
- ☐ False

**B5. The COVID-19 vaccine transforms people into crocodiles?**

- ☐ True
- ☐ False

**B6. The vaccine may cause pain or swelling at injection site or systemic symptoms like fever and or headache**

- ☐ True
- ☐ False

**B7. I will still continue to wear my mask wash my hands or sanitizing and keep a safe distance after receiving the COVID-19 Vaccine?**

- ☐ Yes
- ☐ No

**B8. Which is the best source of information dissemination?**

- ☐ Radio
- ☐ Television
- ☐ Newspaper
- ☐ Social media
- ☐ Internet
- ☐ Other____________________

**B9. Which are your trusted source(s) of information on COVID-19.**

**(You may select more than one option)**

- ☐ Social media
- ☐ Government
- ☐ WHO
- ☐ Internet
- ☐ Other (specify)

*Turn Page Over*

## SECTION C – EXPERIENTIAL FACTORS

**C1. Are you willing to receive the COVID-19 vaccine when is rolled out nationally?**

- [ ] Yes
- [ ] No

**C2. Do you believe that it should be compulsory for people to receive the COVID-19 vaccine?**

- [ ] Yes
- [ ] No

**C3. Do you believe the COVID-19 vaccine is safe for use?**

- [ ] Yes
- [ ] No

**C4. If you will be told that the vaccine may cause swelling or pain at injection site or fever and headache would you still take the vaccine.**

- [ ] Yes
- [ ] No

**C5. Have you ever received any vaccine in the past?**

- [ ] Yes
- [ ] No

**C6. Have you ever refused any vaccine in the past?**

- [ ] Yes
- [ ] No

## SECTION D – SOCIO-CULTURAL FACTORS/NGWAO, SETSO LE TUMELO

**D1. Would your religious or cultural beliefs hinder you from taking the vaccine?**

- [ ] Yes
- [ ] No

**D2. Do you trust other traditional or religious methods of prevention over vaccine?**

- [ ] Yes
- [ ] No

***END***
