# Supplementary material for: Acceptance rate and risk perception towards the COVID-19 vaccine in Botswana
Source: PLoS One. 2022 Feb 4;17(2):e0263375. doi: 10.1371/journal.pone.0263375 (PMC8815939; doi:10.1371/journal.pone.0263375)
Supplement: S2 Questionnaire — (PDF) [file pone.0263375.s003.pdf]

Republic of Botswana
Ministry of Health and Wellness

DIPOTSO
DIPATLISISO KA MAIKUTLO LE KAMOGELO YA MOKENTO WA COVID-19 MO BOTSWANA

SECTION A – DEMOGRAPHIC INFORMATION

A1. Boagedi

☐ Motswana
☐ Tse dingwe: ____________________

A2. Dingwaga ____________________

A3. Bong

☐ Rre
☐ Mme
☐ Tse dingwe

A4. Lenyalo

☐ Ga ke a nyalwa
☐ Ke nyetswe
☐ Ke ne ke nyetswe
☐ Motlholagadi/moswagadi

A5. Lefelo la bonno

☐ Motse selegae
☐ Motse setoropo
☐ Toropo

A6. Tiro

☐ Ke a bereka
☐ Ga ke bereke
☐ Ke moithuti
☐ Tse dingwe

Turn Page Over

A7. Dithuto

- [ ] Primary
- [ ] Junior Secondary
- [ ] BGCSE/ IGCSE
- [ ] Under Graduate
- [ ] Post Graduate

A8. Tumelo

- [ ] Sekeresete
- [ ] Hindu
- [ ] Islam
- [ ] Buddhism
- [ ] Tse dingwe

SECTION B – DIKITSO

B1. A o tshela ka bongwe jwa malwetse a a fa tlase?

- [ ] Madi a Matona
- [ ] Sukiri
- [ ] Kankere
- [ ] Khupelo
- [ ] Malwetse a pelo
- [ ] Malwetse a mahatlha
- [ ] A mangwe ______________________
- [ ] Ga gona

B2. A o itse ka mokento wa COVID-19?

- [ ] Ee
- [ ] Nnyaa

B3. A mokento o le nosi o ka go sireletsa mo bolwetseng jwa COVID-19?

- [ ] Ee
- [ ] Nnyaa

Turn Page Over

B4. Mokento wa COVID-19 o tsewa ke batho ba ba dingwaga tse di lesome le boferabobedi fela.

- [ ] Nnete
- [ ] Ga se nnetea

B5. Mokento wa COVID-19 o fetola batho o ba dira dikwena?

- [ ] Nnete
- [ ] Ga se nnete

B6. Mokento o ka baka ditlamorago tse di akaretsang go ruruga fa go neng go kentiwa teng kgotsa go gotela le tlhogo e opang.

- [ ] Nnete
- [ ] Ga se nnete

B7. Ke tla tswelela ka go rwala mask, go tlhapa diatla le go katologana morago ga go tsaya mokento wa COVID-19.

- [ ] Ee
- [ ] Nnyaa

B8. Ke ofe motlhale o o siameng wa go gasa melaetsa?

- [ ] Seromamowa
- [ ] TV
- [ ] Pampiri ya Dikgang
- [ ] Maranyane
- [ ] Internet
- [ ] Tse Dingwe____________________

B9. Ke efe metswedi ya melaetsa e e tshepegang?

- [ ] Maranyane
- [ ] Goromente
- [ ] WHO
- [ ] Internet
- [ ] Tse Dingwe____________________

Turn Page Over

SECTION C – MAIKUTLO LE MAITEMOGELO

C1. A o ikemiseditse go tsaya mokento wa COVID-19 fa o nna teng?

☐ Ee

☐ Nnyaa

C2. O dumela fa mongwe le mongwe a tshwanetse go tsaya mokento o?

☐ Ee

☐ Nnyaa

C3. A o dumela fa mokento wa COVID-19 o babalesegile go dirisiwa?

☐ Ee

☐ Nnyaa

C4. Fa o ka itse gore mokento o wa COVID-19 o na le ditlamorago (go ruruga fa o kentilweng teng, go gotela le tlhogo), o ka dumela go tswelela o o tsaya?

☐ Ee

☐ Nnyaa

C5. A o kile wa tsaya mokento wa thibelo mo nakong e e fetileng?

☐ Ee

☐ Nnyaa

C6. A o kile wa seka wa dumela go tsaya mokento mo nakong e e fetileng?

☐ Ee

☐ Nnyaa

SECTION D – NGWAO, SETSO LE TUMELO

D1. A o na le mabaka a setso kgotsa a tumelo a ka go itsang go tsaya mokento wa COVID-19?

☐ Ee

☐ Nnyaa

D2. A o dumela gore o na le ditsela tse dingwe tsa tumelo kgotsa ngwao tse di ka go sireletsang go na le mokento wa COVID-19?

☐ Ee

☐ Nnyaa
