# Supplementary material for: Acceptance rate and risk perception towards the COVID-19 vaccine in Botswana
Source: PLoS One. 2022 Feb 4;17(2):e0263375. doi: 10.1371/journal.pone.0263375 (PMC8815939; doi:10.1371/journal.pone.0263375)
Supplement: Attachment — Submitted filename: Response to reviewers.docx [file pone.0263375.s004.docx]

Dr Lebapotswe Bahumi Tlale, MBBS

16^th^ June 2021

David Teye Doku
Academic Editor
PLOS ONE

Dear Sir

**RE: Manuscript ID PONE-D-21-09673 Acceptance Rate and Risk Perception towards the COVID-19 Vaccine in Botswana: A Cross-Sectional Study. PLOS ONE**

Thank you for your and the reviewer’s thoughtful review of our manuscript. With these in mind we are pleased to submit a revised manuscript for publication in the PLOS ONE Journal. The manuscript has not been published, and is not being considered for publication elsewhere.

These are our responses to each point raised by the academic editor and the reviewers:

**Academic Editors Comments:**

**Comment 1**: Please ensure that your manuscript meets PLOS ONE's style requirements, including those for file naming. The PLOS ONE style templates can be found at

http://www.journals.plos.org/plosone/s/file?id=wjVg/PLOSOne_formatting_sample_main_body.pdf and <http://www.journals.plos.org/plosone/s/file?id=ba62/PLOSOne_formatting_sample_title_authors_affiliations.pdf>

Response: Thank you for clarifying this to us. We have formatted the manuscript to meet the PLOS ONE style requirements as advised by the academic editor.

**Comment 2:** Please amend your current ethics statement to include the full name of the ethics committee/institutional review board(s) that approved your specific study.

**Response:** Thank you for highlighting this to us. We have included the following statement in our manuscript. The protocol and any amendments made were submitted to the Botswana, Ministry of Health and Wellness an independent Ethics Committee (EC) or Institutional Review Board (IRB), were it was approved”. We have also attached the approval certificate as part of documents submitted.

**Comment 3:** Please include additional information regarding the survey or questionnaire used in the study and ensure that you have provided sufficient details that others could replicate the analyses. For instance, if you developed a questionnaire as part of this study and it is not under a copyright more restrictive than CC-BY, please include a copy, in both the original language and English, as Supporting Information. Moreover, please include more details on how the questionnaire was pre-tested, and whether it was validated.

**Response:** Thank you for your comment. We will include the copies of the questionnaires both in English and Original language as part of other documents submitted with the manuscript.

**Comment 4**: Thank you for stating the following in the Acknowledgments Section of your manuscript:

[We also thank the FHI Botswana for partially funding this research project.]

We note that you have provided funding information that is not currently declared in your Funding Statement. However, funding information should not appear in the Acknowledgments section or other areas of your manuscript. We will only publish funding information present in the Funding Statement section of the online submission form.

Please remove any funding-related text from the manuscript and let us know how you would like to update your Funding Statement. Currently, your Funding Statement reads as follows:

[The funders had no role in study design, data collection and analysis, decision to publish, or preparation of the manuscript.]

Please provide an amended statement that declares *all* the funding or sources of support (whether external or internal to your organization) received during this study, as detailed online in our guide for authors at http://journals.plos.org/plosone/s/submit-now. Please also include the statement “There was no additional external funding received for this study.” in your updated Funding Statement.

Please include your amended statements within your cover letter; we will change the online submission form on your behalf.

**Response:** Thank you for highlighting this to us and we have removed the statement from the acknowledgement section to read “We also thank the FHI Botswana for also participating in this research project.” We do not wish to change the funding statement and should remain as is. The partial funding that we referred to was payment of publication fees by FHI. They did not fund the research project.

FHI Botswana paid the article publication fee. No additional external funding was received for this study.

**Comment 5:** Thank you for stating the following in the Financial Disclosure section:

[The funders had no role in study design, data collection and analysis, decision to publish, or preparation of the manuscript.]

We note that one or more of the authors are employed by a commercial company: FHI 360

Please provide an amended Funding Statement declaring this commercial affiliation, as well as a statement regarding the Role of Funders in your study. If the funding organization did not play a role in the study design, data collection and analysis, decision to publish, or preparation of the manuscript and only provided financial support in the form of authors' salaries and/or research materials, please review your statements relating to the author contributions, and ensure you have specifically and accurately indicated the role(s) that these authors had in your study. You can update author roles in the Author Contributions section of the online submission form.

Please also include the following statement within your amended Funding Statement.

“The funder provided support in the form of salaries for authors [insert relevant initials], but did not have any additional role in the study design, data collection and analysis, decision to publish, or preparation of the manuscript. The specific roles of these authors are articulated in the ‘author contributions’ section.”

If your commercial affiliation did play a role in your study, please state and explain this role within your updated Funding Statement.

2. Please also provide an updated Competing Interests Statement declaring this commercial affiliation along with any other relevant declarations relating to employment, consultancy, patents, products in development, or marketed products, etc.

Within your Competing Interests Statement, please confirm that this commercial affiliation does not alter your adherence to all PLOS ONE policies on sharing data and materials by including the following statement: "This does not alter our adherence to PLOS ONE policies on sharing data and materials.” (as detailed online in our guide for authors http://journals.plos.org/plosone/s/competing-interests) . If this adherence statement is not accurate and there are restrictions on sharing of data and/or materials, please state these. Please note that we cannot proceed with consideration of your article until this information has been declared.

Please include both an updated Funding Statement and Competing Interests Statement in your cover letter. We will change the online submission form on your behalf.

Please know it is PLOS ONE policy for corresponding authors to declare, on behalf of all authors, all potential competing interests for the purposes of transparency. PLOS defines a competing interest as anything that interferes with, or could reasonably be perceived as interfering with, the full and objective presentation, peer review, editorial decision-making, or publication of research or non-research articles submitted to one of the journals. Competing interests can be financial or non-financial, professional, or personal. Competing interests can arise in relationship to an organization or another person. Please follow this link to our website for more details on competing interests: http://journals.plos.org/plosone/s/competing-interests.

**Response:** Thank you raising this concern. We would like to clearly state that there are no competing interest to declare in this research project. FHI as a commercial organisation is a member of the ministry of health and wellness communication team (Part of the National Public Health Emergency preparedness committee). The author or FHI did not fund and did not play a role in the study design, data collection and analysis, decision to publish, or preparation of the manuscript and also did not provide financial support in the form of authors' salaries and/or research materials for this study. The Author referred above was part of the manuscript write up and did not influence the results, discussion or conclusion of the study.

There was no funding received for this research project and none of the authors received any salaries for undertaking this research project. This applies to all the authors listed in the manuscript.

**Comment 6:** In your Data Availability statement, you have not specified where the minimal data set underlying the results described in your manuscript can be found. PLOS defines a study's minimal data set as the underlying data used to reach the conclusions drawn in the manuscript and any additional data required to replicate the reported study findings in their entirety. All PLOS journals require that the minimal data set be made fully available. For more information about our data policy, please see http://journals.plos.org/plosone/s/data-availability.

Upon re-submitting your revised manuscript, please upload your study’s minimal underlying data set as either Supporting Information files or to a stable, public repository and include the relevant URLs, DOIs, or accession numbers within your revised cover letter. For a list of acceptable repositories, please see http://journals.plos.org/plosone/s/data-availability#loc-recommended-repositories. Any potentially identifying patient information must be fully anonymized.

Important: If there are ethical or legal restrictions to sharing your data publicly, please explain these restrictions in detail. Please see our guidelines for more information on what we consider unacceptable restrictions to publicly sharing data: http://journals.plos.org/plosone/s/data-availability#loc-unacceptable-data-access-restrictions. Note that it is not acceptable for the authors to be the sole named individuals responsible for ensuring data access.

We will update your Data Availability statement to reflect the information you provide in your cover letter.

**Response:** Thank you for highlighting this to us. We will submit our minimal data set with this manuscript as a supporting information file.

**Comment 7:** We note you have included a table to which you do not refer in the text of your manuscript. Please ensure that you refer to Table 2, 7 and 8 in your text; if accepted, production will need this reference to link the reader to the Table.

**Response:** Thank you for bringing this to our attention. We have added some text to describe the above tables

**Comment 8**: Please include a title for Table 4.

**Response:** Thank you for bringing this to our attention. We have included a title for table 4.

**Reviewer’s comments:**

**Reviewer 1 comments:** Thank you for considering me to review this manuscript, “Acceptance Rate and Risk Perception towards the COVID-19 Vaccine in Botswana: Across-Sectional Study”. As the only viable option to mitigate the COVID-19 pandemic is via vaccination, this implies understanding vaccination hesitancy is paramount so that factors that contribute to vaccination hesitancy could be recognized so that effectively counteract measures -health education - could be contemplated. This study purports to explore the acceptance rate and risk perception of COVID-19 vaccines amongst the general population in southern Africa country, Botswana. The authors developed their questions to solicit acceptance rate and risk perception. The authors also included various socio-demographic information and clinical risk factors. The result seems interesting but the authors need to address some issues to render their manuscript with high calibre scientific merit.

**Response:** Thank you for the insights. We will work on the feedback and respond appropriately.

**Comment** •TITLE: The content of the study -the instrument used- is currently not clear to the readers. I would think here that the title would once the outcome measures are made explicit in the method section.

**Response:** Thank you for highlighting this to us. We have worked on our title and instrument used by responding appropriately to the comments made on the method section.

**Comment** •ABSTRACT: The ABSTRACT in general has been structured according to the style of this journal. Minor issues: change “Introduction” to “Background”. During COVID, there is a ‘tradition’ in most journals to include the dates of the data collection (1-28 February 2021).

It seems there is the repetition of the aims (“This study, aims to assess the acceptance rate and risk perception of COVID-19 vaccines amongst the general population in Botswana” … “This study aims to assess the acceptance rate and risk perception of COVID-19 vaccines amongst the general population in Botswana”. SUGGESTION, keep one and create a subheading -Method. In the method, bring outcomes measures on board and the fact the study explored socio-demographic information and some risk factors.

This information is not needed (…” and 31.3% of participants”) (“At P<0.05, the following...”. Suggestion: delete

Word employed should be geared toward the outcome measures of the study.

KEYWORD. This keyword should be included (“vaccine hesitancy”)

**Response:** The comments are all welcome and have been incorporate into the manuscript text. The abstract now reads as follows:

“**Background**: The COVID-19 disease burden continues to be high worldwide and vaccines continue to be developed to help combat the pandemic. Acceptance and risk perception for COVID-19 vaccines is unknown in Botswana despite the government’s decision to roll out the vaccine nationally.

**Objectives**: This study aims to assess the acceptance rate and risk perception of COVID-19 vaccines amongst the general population in Botswana.

**Methods:** We interviewed 5300 adults in Botswana from 1-28 February 2021 using self-administered questionnaires. The main outcomes of the study were vaccine acceptance and hesitancy rates. Demographic, experiential and socio-cultural factors were explored for their association with outcome variables.

**Results:** Two-thirds of the participants were females (3199), with those aged 24-54 making the highest proportion (61%). The acceptance rate of COVID-19 vaccine was 73.4% with vaccine hesitancy at 31.3%. Factors found to be associated with willingness to take the COVID-19 vaccine were sex (p= 0.033), age group (p= 0.009), education level (p= 0.000), willingness to wear face mask (p=0.000), religious beliefs (p=0.000), occupation (p=0.023) and marital status (p=0.045). Safety perception of the vaccine was associated with education level (p= 0.000), willingness to wear face mask (p=0.000), age group (p=0.000) and religion (p=0.001). Ninety six percent (96%) of those willing to receive the vaccine indicated willingness to continue wearing masks and social distancing after vaccination. Radio and Television were the preferred sources for COVID-19 prevention and vaccination information.

**Conclusion**: This study demonstrated a high acceptance rate for the COVID-19 vaccine and a low risk perception in Botswana. In order to achieve a high vaccine coverage and ensure a successful vaccination process, there is need to target populations with high vaccine hesitancy rates. A qualitative study to assess the factors associated with vaccine acceptance and hesitancy is recommended to provide an in-depth analysis of the findings.”

KEYWORDS: Vaccine hesitancy added to the key words. Keywords now read;

“Acceptance Rate, vaccine hesitancy, Risk Perception COVID-19 Vaccine, Case, Contact and Contact Tracing””

**Comment:** •BACKGROUND INFORMATION (INTRODUCTION)

The background information appears to lead the reader to sense what is coming up as the aims of this study -well done.

This statement should be rephrased (“Vaccine acceptance mirrors the public’s perception towards the disease threat, demand for and attitude towards the vaccine”)

The word ‘race’ (“With regards to race, African-Americans and Hispanics demonstrated higher vaccine hesitancy than other races in a US study [16].”) is confusing. Suggestion: ethic group or simple state as following (“There is subcultural diversity, African-Americans and Hispanics demonstrated higher vaccine hesitancy than other cultural or ethnic groups in a US study”.

To avoid too much rambling (“Global literature has also recognized the distinctive roles of several socio-demographic determinants of health in vaccine acceptance and risk perception. Additionally, ….Moreover, credible sources of information about vaccines such as government were reported to instil high levels of trust amongst the public[10].”). Since these paragraphs are preceded by a literature view on the rate of vaccine hesitancy, this paragraph should simply articulate ‘associated factors of vaccine hesitancy’. For a better flow of information, the narration on the rate of vaccine hesitancy should be sent to the previous paragraph.

For aims (“We therefore assessed the acceptance and perceptions of COVID-19 vaccines in Botswana in order to inform the planned population roll out of the vaccines.”), it should reflect the content of the instrument used or developed. Also, the associated factors explored should be mentioned as one of the aims.

**Response: Thank you for this suggestion. We have amended the background as suggested. It now reads:**

“ **Background**

According to the World Health Organisation (WHO) weekly epi update of the 14^th^ February 2021, there were a total of 108 246 992 COVID-19 cases and 2 386 717 COVID-19 deaths [1]. Of these, the African region reported a total of 2 723 431 COVID-19 cases and 68 294 deaths, with Botswana reporting 24 926 total cases and 202 total deaths [1]. Vaccines are considered one of the most awaited interventions for combating COVID-19 and hundreds of global institutions are working at an unprecedented speed to develop COVID-19 vaccines [2-9]. Several vaccines have been developed and some are still undergoing clinical trials while very few countries have started vaccine rollout [10]. One of the challenges towards COVID-19 vaccination is the uncertainty of vaccine acceptance among the public. In general factors that influence vaccine acceptance include the public’s demand for vaccine, their perception towards the disease and attitudes towards the vaccine [11]

Acceptance and perception studies are important as they provide critical information for health education programs to increase uptake of vaccines and target certain populations. Few studies have been conducted assessing the acceptance, perceptions and attitudes towards COVID-19 vaccines as well as factors influencing uptake which may vary from country to country. A global survey of potential acceptance of a COVID-19 vaccine has shown that differences in acceptance rates ranged from almost 90% (in China) to less than 55% (in Russia) [10].In an Australian study [12], eighty per cent (80%) respondents generally held positive views towards COVID-19 vaccination while 65% of participants in Saudi Arabia [2], showed interest to accept the COVID-19 vaccine if available. The preliminary results of a Chilean case study on COVID-19 vaccine perception in the country showed that 87% were willing to vaccinate, a relatively high proportion and slightly lower than the rate found by García and Cerda [13] which was 90.6% for Chile. Gender and age disparities in attitudes were characterised in Australia, where females (83%) were found to be more likely to depict an optimistic outlook to receiving vaccinations than males (78%), as well as those aged 70 years and above (91%) compared to 76% of 18–29-year-olds [12]. Furthermore, willingness to accept future COVID-19 vaccines in Saudi Arabia was relatively high among older age groups, married participants, participants with a postgraduate degree or higher education level (68.8%), non-Saudi (69.1%), employed in government sector (68.9%) [2]. There is subcultural diversity as African Americans and Hispanics demonstrated higher vaccine hesitancy than other cultural or ethnic groups in a US study [16].

Religion, female gender, residing in deprived neighbourhoods were some factors found to be correlated with COVID-19 vaccine hesitancy in Australia (Edwards et al, 2021), while reliance on social media and refraining from news (Chadwick et al, 2021) were also associated with vaccine hesitancy in the UK.

These studies demonstrate significant variations in COVID-19 vaccine acceptance across different countries and the roles of several socio-demographic determinants of health in vaccine acceptance and risk perception. Such information is critical for context and country specific implementation of COVID-19 vaccination programs, with governments being encouraged to understand communities’ concerns and identify strategies that will support engagement to support effective launching of new vaccines [12]

Circumstantial conditions such as the pandemic context, specifically disease prevalence in a particular population can also impact vaccination intention [13]. Perceived severity of COVID-19 and perceived vaccine safety were the two strongest determinants of vaccine acceptance in a Finland study [14]. In Turkey and the UK, acceptance rates of vaccines were found to be higher among study participants who believed in the natural origin of COVID-19 in contrast to those who believed that the disease was generated by humans [15]. Moreover, credible sources of information about vaccines such as government were reported to instil high levels of trust amongst the public [10].

In the African setting, the level of vaccine acceptance (53.6%) and risk perception of (46.7%) were relatively average in Western Uganda [17]. Males, those with a tertiary education, students and non-salary earners were likely to accept the vaccine [17].

The Botswana government has adopted a multi-pronged strategy as part of the response to COVID-19. One of the pillars of the COVID-19 epidemic control measures includes vaccinating 276, 078 (16.5%) of the targeted population in the 1^st^ phase of the vaccination campaign. Botswana is a signatory to the WHO/Worldbank vaccine initiative and expects to receive its first COVID-19 vaccines in March 2021. Acceptance and risk perception for COVID-19 vaccines is unknown in Botswana. There have been several conspiracy theories around COVID-19 vaccines which are mostly linked to religious and cultural beliefs which may influence the uptake of the vaccine [19]. The widely used social media platforms have also reported on the negative aspects of the vaccine and its side effects which may also influence the population’s attitudes and perceptions resulting in low acceptance [20]. We therefore assessed the acceptance and risk perceptions of COVID-19 vaccines in Botswana in order to inform the planned population roll out of the vaccines. Demographic, experiential and socio-cultural factors were also explored for their association with the outcome measures.”

**Comment** •MATERIALS AND METHODS: The problem with the METHOD is that the authors failed to elaborate on the development of the questionnaire used. Further, there is no information on the reliability and other important information on its applicability to the present population. Please attend to this issue.

Overall, the method has much to be desired. I would encourage the authors to stick with the subheadings that are recommended by Strobe observational study checklist (https://www.strobe-statement.org/index.php?id=available-checklists).

This statement touches on an important part of the study (“Following consent, participants were enrolled into the study by administering the questionnaires in the selected localities. This happened at government departments, shops, markets and private companies”.). How data collection was done should be more explicit in the text. Please identify the name of the technique for data collection (probabilistic sampling as stated in the limitation? ).

Different parts of the text describing recruiting and data collection should be consistent with each other. Thus, strict adherence to Strobe observational study checklist would help.

For us international reader, we may not understand what constitute “across the nine (9) COVID-19 zones in Botswana”. Please define.

The rationale for including this statement (“A risk perception model which integrates three core dimensions; cognitive factors (knowledge), experiential factors (emotion) and socio-cultural factors (norms, values) was used to guide in the development of the questions”) is needed. Also, a separate section is needed to highlight the development of the questionnaire. This should be merged with this (“designed from literature review [12, 21, 22]”).

Any reference number was assigned for the present ethical approval from IRB?

The result section has a lot of information and some of them were not described in the methodology or stated as the aim of the study. This is major misgiving of this manuscript. The confusion partly stems from the factor that outcome measures were not described in the method.

Information in different tables should be merged into coherent themes.

**Response:** Thank you for clarifying this to us. We reviewed our methodology section and used the strobe guidelines to write our methods section clearly. The new method section is as below.

“**Materials and Methods**

**Study Design**

This study used a cross sectional survey design conducted across the nine (9) COVID-19 zones in Botswana from 1-28 February 2021 using enumerator administered questionnaires designed from literature review and using a risk perception model which integrates three core dimensions; cognitive factors (knowledge), experiential factors (emotion) and socio-cultural factors (norms, values) [12, 23, 24]. The questionnaires were self-administered without any interference from the interviewer.

**Study Setting**

Botswana is a landlocked country situated in Southern Africa and shares borders with South Africa, Namibia, Zimbabwe and Zambia. The dynamics of population movement across borders with neighbouring countries increases the risk of transmission of communicable diseases including COVID-19. Botswana has an estimated population of 2.374,697, (1,171,629 Males and 1,203,068 Females) as per the 2011 population census projection for 2020 [22]. The population is spread over vast land of 581,730km^2^. The estimated population density in Botswana is 4 per Km. Sixty nine percent (69.4%) of the total population lives in urban settings.

Botswana’s economy is largely dependent on mineral revenues and belongs to Southern Africa Custom Union. The Gross National Income (GNI) per capita is approximately $6,000, thus by classification, Botswana belongs to the upper middle-income countries. Botswana recorded its first COVID-19 case in March 2020. After recording the first case the country was demarcated into nine (9) sections called COVID-19 zones in order to restrict movement of non-essential travel between these zones. In order to travel between these zones, a valid permit, which should be applied for online, is necessary.

**Participants**

The study population consisted of 5300 adults aged 18 years and above since most candidate vaccines were investigated and tried in this group worldwide. In addition, this was in line with the Botswana government policy for COVID-19 vaccine eligibility. Study participants were selected from localities in each of the nine (9) Botswana COVID-19 zones using stratified sampling. Eligibility for participation in the study included those aged 18 years and above including pregnant women, and those with (and without) comorbidities and able to give informed consent. Following consent, participants were enrolled into the study by administering the questionnaires in the selected localities. This happened at government departments, shops, markets and private companies. Exclusion criteria included those who are under 18 years of age and those that did not consent to participate in the study.

**Study Size**

The survey leveraged on the already existing resources. The nine (9) COVID-19 zones were used as strata (Table 1). A stratified sampling was employed for this survey. Individuals/study participants were selected within the localities randomly selected from the COVID-19 zones.

To compute the sample size for the survey a margin of error of 0.05 and a confidence level of 0.95 were used. We further assumed an acceptance rate of 53.6% which is similar to the one estimated by Echoru I et al (2020) in Uganda [19] resulting in a sample of size 383. To cover the 9 COVID-19 zones, we estimated a sample size of 383 multiplied by nine (9) zones giving a total of 3447. To accommodate the design effect, since our sampling strategy is stratified sampling scheme, we assumed a design effect of 1.5 which then gave us a sample size of 5171. Finally, we assumed 90% response rate which resulted in a total sample of **5745** for the survey. **Table 1. The 9 COVID-19 Zones, Sample Size and Allocation**

| Zone | Population | | Proportional Sample | |
| --- | --- | --- | --- | --- |
|  | Localities | >=18 years | Locality | Individuals |
| Greater Gaborone | 167 | 590,689 | 3 | 2712 |
| Greater Palapye | 78 | 188,428 | 2 | 865 |
| Maun | 52 | 84,449 | 1 | 388 |
| Greater Francistown | 80 | 192,483 | 2 | 884 |
| Chobe | 18 | 23,789 | 1 | 109 |
| Ghanzi | 17 | 22,474 | 1 | 103 |
| Kgalagadi | 47 | 30,678 | 1 | 141 |
| Selebi Phikwe | 21 | 74,678 | 1 | 343 |
| Boteti | 20 | 43,810 | 1 | 201 |
| Total | 500 | 1,238,768 | 13 | **5745** |

**Variables**

The exposure variables for this study included age, gender, level of education, Occupation, religious background, Media, marital status and place of residence. The primary outcome measure includes acceptance rate and risk perception towards the COVID-19 vaccine.

**Bias**

Participation was ere selected on a voluntary basis therefore the survey had potential for self-selection bias by community members who are particularly concerned about the pandemic. However, probabilistic sampling was employed. The study also used self-administered questionnaires and this could have an impact on the response rate by the participants.

**Data Sources**

The cross-sectional survey used questionnaires to interview participants from selected areas across the country. The questionnaires were written in both English and Setswana and data obtained from the questionnaire were analysed using the STATA statistical software version 15. The questionnaires were validated before being administered to all the participants. The questionnaires were The participants answered the same questionnaire across zones to obtain reliable information. Data protection was ensured via file password protection and limiting access of information to the project team members only.

**Statistical Methods**

Categorical variables were analysed using summary statistics such as frequencies and percentages. For continuous variables such as age, median (inter-quartile range) or mean (and standard deviation) were presented depending on whether the variable under consideration is skewed or not, respectively. Acceptance was measured by the question asking respondents if they are willing to receive the COVID-19 vaccine when it is rolled out nationally. The acceptance rate of COVID-19 vaccine was the number that “accept” divided by the sample size but excluding those with missing responses. The independent variables were demographic variables such as age, gender, marital status, educational level, and other important variables such presence and absence of comorbidities, COVID-19 zone level (high/red zone, medium, low), socio-cultural and experimental factors. Risk perception was measured by the questions asking respondents if they considered the COVID-19 vaccine to be safe.

Bivariate analysis using the chi-square test was conducted to determine factors associated with both acceptance and risk perception for the COVID-19 vaccine. Multiple logistic regression was conducted to adjust for any confounding factors. Odds ratios, as measures of effect, and the corresponding p-values and 95% confidence intervals are presented. A p-value less than 0.05 was used to determine significance.

**Ethical considerations**

This study was conducted according to Botswana, and International Standards of Good Clinical Practice, applicable government regulations and Institutional research policies and procedures. The protocol and any amendments made were submitted to the Botswana, Ministry of Health and Wellness Institutional Review Board (IRB), were it was approved. The protocol number awarded for this study is HPDME 13/18/1

All subjects were asked to provide written informed consent before responding to questions and they were free to withdraw at any time during the study.”

•**DISCUSSION** I am not going to dwell too much on the discussion section since would invariably be changed if the above-mentioned suggestions are contemplated

**Response:** Thank you for highlighting this to us we have worked on our discussion section to be aligned to the methods and objectives of the study.

•ACKNOWLEDGEMENT

You want to separate acknowledged name (ACKNOWLEDGMENT) person by coma rather than bullets

**Response**: Thank you for highlighting this to us. We have corrected this mistake in the Acknowledgement section

•REFERENCES

The authors have employed 30 references. Most of them are relevant and update.

**Response**: Thank you for this comment. We will continue to use relevant and updated literature.

**Reviewer 2 comments:**

**Comment 1**. General Comments

This is a relevant and current topic. As countries work to start COVID-19 vaccination programmes, the issues of acceptance and risk perception are essential research topics. This is more so in Africa where the epidemiology of COVID-19 has been different and thus risk perception and acceptance of vaccination is likely to differ from developed countries. The manuscript is generally well written.

**Response:** Thank you for the insights. We will work on the feedback and respond appropriately.

Specific Comments:

1. Throughout the manuscript, you use COVID at some places and COVID-19 at other places. Please use COVID-19.

**Response:** Thank you for this clarification. We have revised the whole manuscript and corrected this mistake.

2. Title: “a cross–sectional study” in my opinion does not add anything useful to the title while increasing the length. So my suggestion is to remove that

**Response:** Thank you for highlighting this to us. “a cross–sectional study” removed from title: It now reads; Acceptance Rate and Risk Perception towards the COVID-19 Vaccine in Botswana

3. Abstract: there is repetition of the study objective at the end of the introduction and then the objective parts. I suggest you remove it at the end of the introduction part

**Response:** Thank you for this clarification. The aim has been removed from the end of the introduction part and placed appropriately as advised.

4. At the results part of the abstract you have “At P<0.05, the following factors were associated with willingness to take the COVID vaccine: gender, education level, occupation and COVID zone”.To make it more meaningful in my opinion, kindly consider revising as ‘factors found to be associated with willingness to take the COVID vaccine were gender (p=…..), education level (p=…), occupation (p=…) and COVID zone (p=…..)’. And do put the actual p values

**Response:** Thank you for this clarification. The statement has been revised as suggested and the actual p-values included. It now reads:

“Factors found to be associated with willingness to take the COVID vaccine were gender (p= 0.033), age group (p= 0.009), education level (p= 0.000), willingness to wear face mask (p=0.000) and Religious beliefs (p=0.000)”

5. Then you have “Safety of the vaccine was associated with age group and religion”. Give readers the evidence of the association please, state the p values at least.

**Response:** Thank you for this significant in-sight. The p values have been stated as suggested. The statement now reads:

“Safety perception of the vaccine was associated with education level (p= 0.000), willingness to wear face mask (p=0.000) age (p=0.000) and religion (p=0.001)”

6. You also have “the acceptance rate of COVID-19 vaccine in Botswana was 73.4% and 31.3% of participants perceived the COVID vaccine as unsafe”. What was the 95% confidence intervals for these rates? That will be very useful

**Response:** Thank you for highlighting this to us. The Confidence intervals have been added to the manuscript and the tables converted to pie charts as advised by the reviewer in other comments made to this document. The document now reads:

“Figure 1 shows that out of 5 027 participants, 3689 were willing to take the vaccine resulting in the acceptance rate of the COVID-19 vaccine in Botswana of 73.4% ( CI: 72.2%, 4.6%)

Figure 2 below shows that out of 4784 participants, 1499 (31.3%) (CI: 30.0%, 32.6%) of participants believed that the COVID-19 vaccine was not safe.”

7. Introduction: generally I feel this could be shorter than current length. It has some aspects more sounding likely discussion using many studies from other places. That could be made more concise and therefore reduce the length. Consider this.

**Response:** Thank you for this clarification**.** Some of the statements have been removed from the introduction, while some were compressed to reduce the length and improve the flow. The background now reads:

“**Background**

According to the World Health Organisation (WHO) weekly epi update of the 14^th^ February 2021, there were a total of 108 246 992 COVID-19 cases and 2 386 717 COVID-19 deaths [1]. Of these, the African region reported a total of 2 723 431 COVID-19 cases and 68 294 deaths, with Botswana reporting 24 926 total cases and 202 total deaths [1]. Vaccines are considered one of the most awaited interventions for combating COVID-19 and hundreds of global institutions are working at an unprecedented speed to develop COVID-19 vaccines [2-9]. Several vaccines have been developed and some are still undergoing clinical trials while very few countries have started vaccine rollout [10]. One of the challenges towards COVID-19 vaccination is the uncertainty of vaccine acceptance among the public. In general factors that influence vaccine acceptance include the public’s demand for vaccine, their perception towards the disease and attitudes towards the vaccine [11]

Acceptance and perception studies are important as they provide critical information for health education programs to increase uptake of vaccines and target certain populations. Few studies have been conducted assessing the acceptance, perceptions and attitudes towards COVID-19 vaccines as well as factors influencing uptake which may vary from country to country. A global survey of potential acceptance of a COVID-19 vaccine has shown that differences in acceptance rates ranged from almost 90% (in China) to less than 55% (in Russia) [10].In an Australian study [12], eighty per cent (80%) respondents generally held positive views towards COVID-19 vaccination while 65% of participants in Saudi Arabia [2], showed interest to accept the COVID-19 vaccine if available. The preliminary results of a Chilean case study on COVID-19 vaccine perception in the country showed that 87% were willing to vaccinate, a relatively high proportion and slightly lower than the rate found by García and Cerda [13] which was 90.6% for Chile. Gender and age disparities in attitudes were characterised in Australia, where females (83%) were found to be more likely to depict an optimistic outlook to receiving vaccinations than males (78%), as well as those aged 70 years and above (91%) compared to 76% of 18–29-year-olds [12]. Furthermore, willingness to accept future COVID-19 vaccines in Saudi Arabia was relatively high among older age groups, married participants, participants with a postgraduate degree or higher education level (68.8%), non-Saudi (69.1%), employed in government sector (68.9%) [2]. There is subcultural diversity as African Americans and Hispanics demonstrated higher vaccine hesitancy than other cultural or ethnic groups in a US study [16].

Religion, female gender, residing in deprived neighbourhoods were some factors found to be correlated with COVID-19 vaccine hesitancy in Australia (Edwards et al, 2021), while reliance on social media and refraining from news (Chadwick et al, 2021) were also associated with vaccine hesitancy in the UK.

These studies demonstrate significant variations in COVID-19 vaccine acceptance across different countries and the roles of several socio-demographic determinants of health in vaccine acceptance and risk perception. Such information is critical for context and country specific implementation of COVID-19 vaccination programs, with governments being encouraged to understand communities’ concerns and identify strategies that will support engagement to support effective launching of new vaccines [12]

Circumstantial conditions such as the pandemic context, specifically disease prevalence in a particular population can also impact vaccination intention [13]. Perceived severity of COVID-19 and perceived vaccine safety were the two strongest determinants of vaccine acceptance in a Finland study [14]. In Turkey and the UK, acceptance rates of vaccines were found to be higher among study participants who believed in the natural origin of COVID-19 in contrast to those who believed that the disease was generated by humans [15]. Moreover, credible sources of information about vaccines such as government were reported to instil high levels of trust amongst the public [10].

In the African setting, the level of vaccine acceptance (53.6%) and risk perception of (46.7%) were relatively average in Western Uganda [17]. Males, those with a tertiary education, students and non-salary earners were likely to accept the vaccine [17].

The Botswana government has adopted a multi-pronged strategy as part of the response to COVID-19. One of the pillars of the COVID-19 epidemic control measures includes vaccinating 276, 078 (16.5%) of the targeted population in the 1^st^ phase of the vaccination campaign. Botswana is a signatory to the WHO/Worldbank vaccine initiative and expects to receive its first COVID-19 vaccines in March 2021. Acceptance and risk perception for COVID-19 vaccines is unknown in Botswana. There have been several conspiracy theories around COVID-19 vaccines which are mostly linked to religious and cultural beliefs which may influence the uptake of the vaccine [19]. The widely used social media platforms have also reported on the negative aspects of the vaccine and its side effects which may also influence the population’s attitudes and perceptions resulting in low acceptance [20]. We therefore assessed the acceptance and risk perceptions of COVID-19 vaccines in Botswana in order to inform the planned population roll out of the vaccines. Demographic, experiential and socio-cultural factors were also explored for their association with the outcome measures.”

8. Study population: you just mentioned that stratified sampling was used to select study participants but offer on more details. How was this done exactly? Were the population size for each of the zones factored into the number recruited per zone? You do not tell us how the 9 zones were created and how that related to the study design.

**Response:** Thank you for highlighting this to us. We have reviewed our Methods section and used Strobe guidelines to re-write this section so that it is well understood. We have also included a table that shows how this stratified sampling was done and number recruited per each Zone. The new sections reads

“Study Size

The survey leveraged on the already existing resources. The nine (9) COVID-19 zones were used as strata (Table 1). A stratified sampling was employed for this survey. Individuals/study participants were selected within the localities randomly selected from the COVID-19 zones.

To compute the sample size for the survey a margin of error of 0.05 and a confidence level of 0.95 were used. We further assumed an acceptance rate of 53.6% which is similar to the one estimated by Echoru I et al (2020) in Uganda [19] resulting in a sample of size 383. To cover the 9 COVID-19 zones, we estimated a sample size of 383 multiplied by nine (9) zones giving a total of 3447. To accommodate the design effect, since our sampling strategy is stratified sampling scheme, we assumed a design effect of 1.5 which then gave us a sample size of 5171. Finally, we assumed 90% response rate which resulted in a total sample of **5745** for the survey.

**Table 1. The 9 COVID-19 Zones, Sample Size and Allocation**

| Zone | Population | | Proportional Sample | |
| --- | --- | --- | --- | --- |
|  | Localities | >=18 years | Locality | Individuals |
| Greater Gaborone | 167 | 590,689 | 3 | 2712 |
| Greater Palapye | 78 | 188,428 | 2 | 865 |
| Maun | 52 | 84,449 | 1 | 388 |
| Greater Francistown | 80 | 192,483 | 2 | 884 |
| Chobe | 18 | 23,789 | 1 | 109 |
| Ghanzi | 17 | 22,474 | 1 | 103 |
| Kgalagadi | 47 | 30,678 | 1 | 141 |
| Selebi Phikwe | 21 | 74,678 | 1 | 343 |
| Boteti | 20 | 43,810 | 1 | 201 |
| Total | 500 | 1,238,768 | 13 | **5745** |

9. Then you say “Individuals/study participants were selected within the localities randomly selected from the Covid-19 zones”, how was this done?

**Response:** Thank you for this clarification. This comment has also been in addressed in comment number 8 above which details how the stratified sampling was done.

10. Was there any exclusion criteria

**Response:** Thank you for this highlight. There was inclusion criteria and we added the exclusion criteria in the manuscript and it reads

“The study population consisted of 5300 adults aged 18 years and above since most candidate vaccines were investigated and tried in this group worldwide. In addition, this was in line with the Botswana government policy for COVID-19 vaccine eligibility. Study participants were selected from localities in each of the nine (9) Botswana COVID-19 zones using stratified sampling. Eligibility for participation in the study included those aged 18 years and above including pregnant women, and those with (and without) comorbidities and able to give informed consent. Following consent, participants were enrolled into the study by administering the questionnaires in the selected localities. This happened at government departments, shops, markets and private companies. Exclusion criteria included those who are under 18 years of age and those that did not consent to participate in the study.”

11. You have “To cover the 9 COVID-19 zones, we estimated a sample of 383 multiplied by giving a total of 3447”, there is something missing. It does not read well to me, kindly have a look.

**Response:** Thank you for this insightful comment. We have noted this mistake and corrected it. The statement now reads as follows

“To cover the 9 COVID-19 zones, we estimated a sample size of 383 multiplied by nine (9) zones giving a total of 3447. To accommodate the design effect, since our sampling strategy is stratified sampling scheme, we assumed a design effect of 1.5 which then gave us a sample size of 5171. Finally, we assumed 90% response rate which resulted in a total sample of 5745 for the survey.”

12. Since this was in-person interviewer administered questionnaire, how was the safety of everyone involved assured?

**Response**: Thank you for this clarification. We have corrected the statement. This were self-administered questionnaires.

13. Results: if this was interviewer administered why do you have so many missing values for almost each of the variables?

**Response:** Thank you for insightful comment. We have noted the error made in the manuscript. This has been corrected in the manuscript, Questionnaires were self-administered.

14. Consider presenting table 2 and 4 as figures rather, I think they will be nicer e.g. as a pie chart or other appropriate figure type. And most importantly, please indicate the 95%CI for the acceptance rates

**Response**: Thank you for this insightful comment. We have added 95%CI and also converted the tables into pie charts as advised.

15. You have just too many tables. Apart from those I have pointed out can be figures, there are still a number of tables that can be put together as 1 larger table and will still be clear. Have a look and do this for tables 6-8 and then 9-10.

**Response**: Thank you for this insightful comments: we have converted some of the tables as advised to figures to reduce the number of tables. We have also merged table 6 and 7 to make one table to reduce the number of table in the manuscript as advised.

16. You mentioned multivariate analysis but I see no such results presented

**Response:** Thank you for highlighting this to us. We have revised our results section and added more analysis to the tables and figures.

17. Check and deal with some few long sentences and grammatical errors.

**Response:** thank you for this clarification. We have reviewed the whole document and dealt with the grammatical errors identified in the manuscript.

**Reviewer 3 Comments.** I have some comments about the manuscript regarding the content of tables, result part, and some minor language issues that follow below. I would strongly recommend that the authors recheck the numbers in the tables.

Results

1) It seems that an error exists in Table 1, page 11 regarding total number of “education level” which is 5310?! Why not 5300?

**Response:** Thank you for highlighting this to us. Table 1 has been revised and the figures has been corrected.

2) In Table 2, page 12: why total number is not 5300? If missing data exist, I suggest that the authors add them to the table

**Response:** Thank you for this clarification. We have noted this mistake and this was because of missing data. The table has now been correct to include missing data.

3) Page 12, the part d of the results about “Factors associated with acceptance for the COVID vaccine in Botswana”: as all the factors have been explained, a short explanation about how occupations were associated with acceptance of the COVID vaccine is good to be added to the text

**Response:** Thank you for this insightful comment. A new and simple Table 3 has been added and the interpretation is also provided.

4) Regarding Table 4, page 13, as total number is 4784, whether 516 is missing here? Then it is better to provide info about missing data in the table

**Response:** thank you for this clarification. We have noted this error and the number has been fixed and the Table numbers has been revised.

5) Regarding Table 6, page 14, are there missing data? then it is better to provide the number of missing data in the table

**Response:** Thank you for highlighting this to us. The error was noted and the number has been fixed and the Table numbers has been revised.

6) Regarding Table 7, page 15, I think it is better to add info about missing data if exist

**Response:** Thank you for highlighting this to us. The number has been fixed and the Table numbers has been revised**.**

7) In Table 8: it seems that an error exists regarding total numbers: 1172+3772

**Response:** Thank you for highlighting this to us. The error has been noted and the number has been fixed and the Table numbers has been revised.

8) In Table 9, page 16: it seems that an error exists here. Regarding total frequencies, why is it 5931? and percent which is 111,9 ?!

**Response:** Thank you for highlighting this to us. The error has been noted and the number has been fixed and the Table numbers has been revised.

9) Table 10: It seems that an error exists here too regarding total frequencies: why is it 6203? and percent which is 117 ?!

**Response:** Thank you for highlighting this to us. The mistake has been noted and the number has been fixed and the Table numbers has been revised.

*And some minor language issues:

Page 15 line 2: Forty nine percent of participants who said their religion and “cultural” hinders vaccine uptake were willing to receive the COVID-19 vaccine. use the word "culture" instead of cultural

**Response:** thank you for the insightful comment. We have done as advised and the new statement reads as follows “Willingness to take the Vaccine against religion and culture”

Regarding title of Table 7, delete the first “believes” and add "religious" instead of religion

**Response:** thank you for this insightful comment: We have noted this and have corrected it to read as follows” Participants whose religious beliefs did not hinder vaccination were more likely to take the vaccine than those whose religious beliefs hinder vaccination. Table 47 shows that almost about half (49%) of the participants who said their religious and cultural beliefs hinder vaccine uptake were willing to receive the COVID-19 vaccine”

Page 15: regarding the line that follows “Willing to receive COVID vaccine though trust other traditional and religious methods over vaccine” delete i

Response: Thank you for this comment. We have corrected the mistake

**Reviewer 4 comments:** Thank you for considering me to review this manuscript, “Acceptance Rate and Risk Perception towards the COVID-19 Vaccine in Botswana: Across-Sectional Study”. As the only viable option to mitigate the COVID-19 pandemic is via vaccination, this implies understanding vaccination hesitancy is paramount so that factors that contribute to vaccination hesitancy could be recognized so that effectively counteract measures -health education - could be contemplated. This study purports to explore the acceptance rate and risk perception of COVID-19 vaccines amongst the general population in southern Africa country, Botswana. The authors developed their questions to solicit acceptance rate and risk perception. The authors also included various socio-demographic information and clinical risk factors. The result seems interesting but the authors need to address some issues to render their manuscript with high calibre scientific merit.

**Response:** Thank you for the insights. We will work on the feedback and respond appropriately.

**Comment** •TITLE: The content of the study -the instrument used- is currently not clear to the readers. I would think here that the title would once the outcome measures are made explicit in the method section.

**Response:** Thank you for highlighting this to us. We have worked on our title and instrument used by responding appropriately to the comments made on the method section.

**Comment** •ABSTRACT: The ABSTRACT in general has been structured according to the style of this journal. Minor issues: change “Introduction” to “Background”. During COVID, there is a ‘tradition’ in most journals to include the dates of the data collection (1-28 February 2021).

It seems there is the repetition of the aims (“This study, aims to assess the acceptance rate and risk perception of COVID-19 vaccines amongst the general population in Botswana” … “This study aims to assess the acceptance rate and risk perception of COVID-19 vaccines amongst the general population in Botswana”. SUGGESTION, keep one and create a subheading -Method. In the method, bring outcomes measures on board and the fact the study explored socio-demographic information and some risk factors.

This information is not needed (…” and 31.3% of participants”) (“At P<0.05, the following...”. Suggestion: delete

Word employed should be geared toward the outcome measures of the study.

KEYWORD. This keyword should be included (“vaccine hesitancy”)

**Response:** The comments are all welcome and have been incorporate into the manuscript text. The abstract now reads as follows:

“**Background**: The COVID-19 disease burden continues to be high worldwide and vaccines continue to be developed to help combat the pandemic. Acceptance and risk perception for COVID-19 vaccines is unknown in Botswana despite the government’s decision to roll out the vaccine nationally.

**Objectives**: This study aims to assess the acceptance rate and risk perception of COVID-19 vaccines amongst the general population in Botswana.

**Methods:** We interviewed 5300 adults in Botswana from 1-28 February 2021 using self-administered questionnaires. The main outcomes of the study were vaccine acceptance and hesitancy rates. Demographic, experiential and socio-cultural factors were explored for their association with outcome variables.

**Results:** Two-thirds of the participants were females (3199), with those aged 24-54 making the highest proportion (61%). The acceptance rate of COVID-19 vaccine was 73.4% with vaccine hesitancy at 31.3%. Factors found to be associated with willingness to take the COVID-19 vaccine were sex (p= 0.033), age group (p= 0.009), education level (p= 0.000), willingness to wear face mask (p=0.000), religious beliefs (p=0.000), occupation (p=0.023) and marital status (p=0.045). Safety perception of the vaccine was associated with education level (p= 0.000), willingness to wear face mask (p=0.000), age group (p=0.000) and religion (p=0.001). Ninety six percent (96%) of those willing to receive the vaccine indicated willingness to continue wearing masks and social distancing after vaccination. Radio and Television were the preferred sources for COVID-19 prevention and vaccination information.

**Conclusion**: This study demonstrated a high acceptance rate for the COVID-19 vaccine and a low risk perception in Botswana. In order to achieve a high vaccine coverage and ensure a successful vaccination process, there is need to target populations with high vaccine hesitancy rates. A qualitative study to assess the factors associated with vaccine acceptance and hesitancy is recommended to provide an in-depth analysis of the findings.”

KEYWORDS: Vaccine hesitancy added to the key words. Keywords now read;

“Acceptance Rate, vaccine hesitancy, Risk Perception COVID-19 Vaccine, Case, Contact and Contact Tracing””

**Comment:** •BACKGROUND INFORMATION (INTRODUCTION)

The background information appears to lead the reader to sense what is coming up as the aims of this study -well done.

This statement should be rephrased (“Vaccine acceptance mirrors the public’s perception towards the disease threat, demand for and attitude towards the vaccine”)

The word ‘race’ (“With regards to race, African-Americans and Hispanics demonstrated higher vaccine hesitancy than other races in a US study [16].”) is confusing. Suggestion: ethic group or simple state as following (“There is subcultural diversity, African-Americans and Hispanics demonstrated higher vaccine hesitancy than other cultural or ethnic groups in a US study”.

To avoid too much rambling (“Global literature has also recognized the distinctive roles of several socio-demographic determinants of health in vaccine acceptance and risk perception. Additionally, ….Moreover, credible sources of information about vaccines such as government were reported to instil high levels of trust amongst the public[10].”). Since these paragraphs are preceded by a literature view on the rate of vaccine hesitancy, this paragraph should simply articulate ‘associated factors of vaccine hesitancy’. For a better flow of information, the narration on the rate of vaccine hesitancy should be sent to the previous paragraph.

For aims (“We therefore assessed the acceptance and perceptions of COVID-19 vaccines in Botswana in order to inform the planned population roll out of the vaccines.”), it should reflect the content of the instrument used or developed. Also, the associated factors explored should be mentioned as one of the aims.

**Response: Thank you for this suggestion. We have amended the background as suggested. It now reads:**

“ **Background**

According to the World Health Organisation (WHO) weekly epi update of the 14^th^ February 2021, there were a total of 108 246 992 COVID-19 cases and 2 386 717 COVID-19 deaths [1]. Of these, the African region reported a total of 2 723 431 COVID-19 cases and 68 294 deaths, with Botswana reporting 24 926 total cases and 202 total deaths [1]. Vaccines are considered one of the most awaited interventions for combating COVID-19 and hundreds of global institutions are working at an unprecedented speed to develop COVID-19 vaccines [2-9]. Several vaccines have been developed and some are still undergoing clinical trials while very few countries have started vaccine rollout [10]. One of the challenges towards COVID-19 vaccination is the uncertainty of vaccine acceptance among the public. In general factors that influence vaccine acceptance include the public’s demand for vaccine, their perception towards the disease and attitudes towards the vaccine [11]

Acceptance and perception studies are important as they provide critical information for health education programs to increase uptake of vaccines and target certain populations. Few studies have been conducted assessing the acceptance, perceptions and attitudes towards COVID-19 vaccines as well as factors influencing uptake which may vary from country to country. A global survey of potential acceptance of a COVID-19 vaccine has shown that differences in acceptance rates ranged from almost 90% (in China) to less than 55% (in Russia) [10].In an Australian study [12], eighty per cent (80%) respondents generally held positive views towards COVID-19 vaccination while 65% of participants in Saudi Arabia [2], showed interest to accept the COVID-19 vaccine if available. The preliminary results of a Chilean case study on COVID-19 vaccine perception in the country showed that 87% were willing to vaccinate, a relatively high proportion and slightly lower than the rate found by García and Cerda [13] which was 90.6% for Chile. Gender and age disparities in attitudes were characterised in Australia, where females (83%) were found to be more likely to depict an optimistic outlook to receiving vaccinations than males (78%), as well as those aged 70 years and above (91%) compared to 76% of 18–29-year-olds [12]. Furthermore, willingness to accept future COVID-19 vaccines in Saudi Arabia was relatively high among older age groups, married participants, participants with a postgraduate degree or higher education level (68.8%), non-Saudi (69.1%), employed in government sector (68.9%) [2]. There is subcultural diversity as African Americans and Hispanics demonstrated higher vaccine hesitancy than other cultural or ethnic groups in a US study [16].

Religion, female gender, residing in deprived neighbourhoods were some factors found to be correlated with COVID-19 vaccine hesitancy in Australia (Edwards et al, 2021), while reliance on social media and refraining from news (Chadwick et al, 2021) were also associated with vaccine hesitancy in the UK.

These studies demonstrate significant variations in COVID-19 vaccine acceptance across different countries and the roles of several socio-demographic determinants of health in vaccine acceptance and risk perception. Such information is critical for context and country specific implementation of COVID-19 vaccination programs, with governments being encouraged to understand communities’ concerns and identify strategies that will support engagement to support effective launching of new vaccines [12]

Circumstantial conditions such as the pandemic context, specifically disease prevalence in a particular population can also impact vaccination intention [13]. Perceived severity of COVID-19 and perceived vaccine safety were the two strongest determinants of vaccine acceptance in a Finland study [14]. In Turkey and the UK, acceptance rates of vaccines were found to be higher among study participants who believed in the natural origin of COVID-19 in contrast to those who believed that the disease was generated by humans [15]. Moreover, credible sources of information about vaccines such as government were reported to instil high levels of trust amongst the public [10].

In the African setting, the level of vaccine acceptance (53.6%) and risk perception of (46.7%) were relatively average in Western Uganda [17]. Males, those with a tertiary education, students and non-salary earners were likely to accept the vaccine [17].

The Botswana government has adopted a multi-pronged strategy as part of the response to COVID-19. One of the pillars of the COVID-19 epidemic control measures includes vaccinating 276, 078 (16.5%) of the targeted population in the 1^st^ phase of the vaccination campaign. Botswana is a signatory to the WHO/Worldbank vaccine initiative and expects to receive its first COVID-19 vaccines in March 2021. Acceptance and risk perception for COVID-19 vaccines is unknown in Botswana. There have been several conspiracy theories around COVID-19 vaccines which are mostly linked to religious and cultural beliefs which may influence the uptake of the vaccine [19]. The widely used social media platforms have also reported on the negative aspects of the vaccine and its side effects which may also influence the population’s attitudes and perceptions resulting in low acceptance [20]. We therefore assessed the acceptance and risk perceptions of COVID-19 vaccines in Botswana in order to inform the planned population roll out of the vaccines. Demographic, experiential and socio-cultural factors were also explored for their association with the outcome measures.”

**Comment** •MATERIALS AND METHODS: The problem with the METHOD is that the authors failed to elaborate on the development of the questionnaire used. Further, there is no information on the reliability and other important information on its applicability to the present population. Please attend to this issue.

Overall, the method has much to be desired. I would encourage the authors to stick with the subheadings that are recommended by Strobe observational study checklist (https://www.strobe-statement.org/index.php?id=available-checklists).

This statement touches on an important part of the study (“Following consent, participants were enrolled into the study by administering the questionnaires in the selected localities. This happened at government departments, shops, markets and private companies”.). How data collection was done should be more explicit in the text. Please identify the name of the technique for data collection (probabilistic sampling as stated in the limitation? ).

Different parts of the text describing recruiting and data collection should be consistent with each other. Thus, strict adherence to Strobe observational study checklist would help.

For us international reader, we may not understand what constitute “across the nine (9) COVID-19 zones in Botswana”. Please define.

The rationale for including this statement (“A risk perception model which integrates three core dimensions; cognitive factors (knowledge), experiential factors (emotion) and socio-cultural factors (norms, values) was used to guide in the development of the questions”) is needed. Also, a separate section is needed to highlight the development of the questionnaire. This should be merged with this (“designed from literature review [12, 21, 22]”).

Any reference number was assigned for the present ethical approval from IRB?

The result section has a lot of information and some of them were not described in the methodology or stated as the aim of the study. This is major misgiving of this manuscript. The confusion partly stems from the factor that outcome measures were not described in the method.

Information in different tables should be merged into coherent themes.

**Response:** Thank you for clarifying this to us. We reviewed our methodology section and used the strobe guidelines to write our methods section clearly. The new method section is as below.

“**Materials and Methods**

**Study Design**

This study used a cross sectional survey design conducted across the nine (9) COVID-19 zones in Botswana from 1-28 February 2021 using enumerator administered questionnaires designed from literature review and using a risk perception model which integrates three core dimensions; cognitive factors (knowledge), experiential factors (emotion) and socio-cultural factors (norms, values) [12, 23, 24]. The questionnaires were self-administered without any interference from the interviewer.

**Study Setting**

Botswana is a landlocked country situated in Southern Africa and shares borders with South Africa, Namibia, Zimbabwe and Zambia. The dynamics of population movement across borders with neighbouring countries increases the risk of transmission of communicable diseases including COVID-19. Botswana has an estimated population of 2.374,697, (1,171,629 Males and 1,203,068 Females) as per the 2011 population census projection for 2020 [22]. The population is spread over vast land of 581,730km^2^. The estimated population density in Botswana is 4 per Km. Sixty nine percent (69.4%) of the total population lives in urban settings.

Botswana’s economy is largely dependent on mineral revenues and belongs to Southern Africa Custom Union. The Gross National Income (GNI) per capita is approximately $6,000, thus by classification, Botswana belongs to the upper middle-income countries. Botswana recorded its first COVID-19 case in March 2020. After recording the first case the country was demarcated into nine (9) sections called COVID-19 zones in order to restrict movement of non-essential travel between these zones. In order to travel between these zones, a valid permit, which should be applied for online, is necessary.

**Participants**

The study population consisted of 5300 adults aged 18 years and above since most candidate vaccines were investigated and tried in this group worldwide. In addition, this was in line with the Botswana government policy for COVID-19 vaccine eligibility. Study participants were selected from localities in each of the nine (9) Botswana COVID-19 zones using stratified sampling. Eligibility for participation in the study included those aged 18 years and above including pregnant women, and those with (and without) comorbidities and able to give informed consent. Following consent, participants were enrolled into the study by administering the questionnaires in the selected localities. This happened at government departments, shops, markets and private companies. Exclusion criteria included those who are under 18 years of age and those that did not consent to participate in the study.

**Study Size**

The survey leveraged on the already existing resources. The nine (9) COVID-19 zones were used as strata (Table 1). A stratified sampling was employed for this survey. Individuals/study participants were selected within the localities randomly selected from the COVID-19 zones.

To compute the sample size for the survey a margin of error of 0.05 and a confidence level of 0.95 were used. We further assumed an acceptance rate of 53.6% which is similar to the one estimated by Echoru I et al (2020) in Uganda [19] resulting in a sample of size 383. To cover the 9 COVID-19 zones, we estimated a sample size of 383 multiplied by nine (9) zones giving a total of 3447. To accommodate the design effect, since our sampling strategy is stratified sampling scheme, we assumed a design effect of 1.5 which then gave us a sample size of 5171. Finally, we assumed 90% response rate which resulted in a total sample of **5745** for the survey. **Table 1. The 9 COVID-19 Zones, Sample Size and Allocation**

| Zone | Population | | Proportional Sample | |
| --- | --- | --- | --- | --- |
|  | Localities | >=18 years | Locality | Individuals |
| Greater Gaborone | 167 | 590,689 | 3 | 2712 |
| Greater Palapye | 78 | 188,428 | 2 | 865 |
| Maun | 52 | 84,449 | 1 | 388 |
| Greater Francistown | 80 | 192,483 | 2 | 884 |
| Chobe | 18 | 23,789 | 1 | 109 |
| Ghanzi | 17 | 22,474 | 1 | 103 |
| Kgalagadi | 47 | 30,678 | 1 | 141 |
| Selebi Phikwe | 21 | 74,678 | 1 | 343 |
| Boteti | 20 | 43,810 | 1 | 201 |
| Total | 500 | 1,238,768 | 13 | **5745** |

**Variables**

**The exposure variables for this study included age, gender, level of education, Occupation, religious background, Media, marital status and place of residence. The primary outcome measure includes acceptance rate and risk perception towards the COVID-19 vaccine.**

**Bias**

**Participation was ere selected on a voluntary basis therefore the survey had potential for self-selection bias by community members who are particularly concerned about the pandemic. However, probabilistic sampling was employed. The study also used self-administered questionnaires and this could have an impact on the response rate by the participants.**

**Data Sources**

The cross-sectional survey used questionnaires to interview participants from selected areas across the country. The questionnaires were written in both English and Setswana and data obtained from the questionnaire were analysed using the STATA statistical software version 15. The questionnaires were validated before being administered to all the participants. The questionnaires were The participants answered the same questionnaire across zones to obtain reliable information. Data protection was ensured via file password protection and limiting access of information to the project team members only.

**Statistical Methods**

Categorical variables were analysed using summary statistics such as frequencies and percentages. For continuous variables such as age, median (inter-quartile range) or mean (and standard deviation) were presented depending on whether the variable under consideration is skewed or not, respectively. Acceptance was measured by the question asking respondents if they are willing to receive the COVID-19 vaccine when it is rolled out nationally. The acceptance rate of COVID-19 vaccine was the number that “accept” divided by the sample size but excluding those with missing responses. The independent variables were demographic variables such as age, gender, marital status, educational level, and other important variables such presence and absence of comorbidities, COVID-19 zone level (high/red zone, medium, low), socio-cultural and experimental factors. Risk perception was measured by the questions asking respondents if they considered the COVID-19 vaccine to be safe.

Bivariate analysis using the chi-square test was conducted to determine factors associated with both acceptance and risk perception for the COVID-19 vaccine. Multiple logistic regression was conducted to adjust for any confounding factors. Odds ratios, as measures of effect, and the corresponding p-values and 95% confidence intervals are presented. A p-value less than 0.05 was used to determine significance.

**Ethical considerations**

This study was conducted according to Botswana, and International Standards of Good Clinical Practice, applicable government regulations and Institutional research policies and procedures. The protocol and any amendments made were submitted to the Botswana, Ministry of Health and Wellness Institutional Review Board (IRB), were it was approved. The protocol number awarded for this study is HPDME 13/18/1

All subjects were asked to provide written informed consent before responding to questions and they were free to withdraw at any time during the study.”

•**DISCUSSION** I am not going to dwell too much on the discussion section since would invariably be changed if the above-mentioned suggestions are contemplated

**Response:** Thank you for highlighting this to us we have worked on our discussion section to be aligned to the methods and objectives of the study.

•ACKNOWLEDGEMENT

You want to separate acknowledged name (ACKNOWLEDGMENT) person by coma rather than bullets

**Response**: Thank you for highlighting this to us. We have corrected this mistake in the Acknowledgement section

•REFERENCES

The authors have employed 30 references. Most of them are relevant and update.

Response: Thank you for this comment. We will continue to use relevant and updated literature.

All authors have seen and approved the manuscript, and have contributed to the work. There are no copyright restrictions. No authors have conflicts of interest to disclose.

Thank you for considering our revised manuscript. Please contact me for additional information.

Sincerely,

Lebapotswe B Tlale

Public Health Specialist

Ministry of health and Wellness

Cell: +267 71416607 and +267 73340732

Email: cypro330@yahoo.com
