# Supplementary material for: Acceptance rate and risk perception towards the COVID-19 vaccine in Botswana
Source: PLoS One. 2022 Feb 4;17(2):e0263375. doi: 10.1371/journal.pone.0263375 (PMC8815939; doi:10.1371/journal.pone.0263375)
Supplement: Attachment — Submitted filename: Response to reviewers.docx [file pone.0263375.s005.docx]

Dr Lebapotswe Bahumi Tlale, MBBS

15^th^ September 2021

David Teye Doku
Academic Editor
PLOS ONE

Dear Sir

**RE: Manuscript ID PONE-D-21-09673 Acceptance Rate and Risk Perception towards the COVID-19 Vaccine in Botswana: A Cross-Sectional Study. PLOS ONE**

Thank you for your and the reviewer’s thoughtful review of our manuscript. With these in mind we are pleased to submit a revised manuscript for publication in the PLOS ONE Journal. The manuscript has not been published, and is not being considered for publication elsewhere.

These are our responses to each point raised by the academic editor and the reviewers:

**Reviewer #1:**

**Comment 1:** Thank you for considering me to re-review this manuscript and now entitled, “Acceptance Rate and Risk Perception towards the COVID-19 Vaccine in Botswana. This manuscript was scrutinized by 3 reviewers including myself. The authors have addressed all the comments raised by the reviewers. Both the conceptual and scientific merit of the manuscript has now improved some minor issues.

**Response:** Thank you for reviewing our manuscript and highlighting the minor issues to be addressed

**Comment 2:** Under “Qualifications and emails of all authors”, some of the authors were not accompanied their qualifications. Any reason?

**Response:** Thank you for highlighting this to us. We have edited this section to include all the authors’ qualification. The revised version is as follows:

“Qualifications and emails of all authors:

Lebapotswe B. Tlale, MBBS (UWI), MMed Public Health UB)	cypro330@yahoo.com

Lesego Gabaitiri	BA, MSc, PhD	gabaitiril@biust.ac.bw

Lorato Totolo Diploma, BA community health, PGD	kaone.regoeng@gmail.com

Gomolemo Smith, MBBS (UB)	gomolemodisang@yahoo.com

Orapeleng Puswane-Katse, MBBS (UWI) MMed (UB)	ophuswane@yahoo.co.uk

Eunice Ramonna, BA social work (UB), MPH (Pretoria)	lapoeunx@gmail.com

Basego Mothowaeng, BA Communication Science	basego07@gmail.com

John Tlhakanelo, MBCHB, MMed (UB)	tlhakaneloj@ub.ac.bw

Tiny Masupe MBCHB, MSC, MPH	tiny.masupe@mopipi.ub.bw

Goabaone Rankgoane-Pono, MBCHB (UPT), MMeD (UB), Dip HE (UCT)goaba2000@yahoo.com

John Irige, MPH	jirige@fhi360.org

Faith Mafa, BA media production	faithmorwaagole@gmail.com

Samuel Kolane, BPH Health Education	[sinki.kolane@gmail.com](mailto:sinki.kolane@gmail.com)”

**Comment 3:** In the method, maybe this statement (“Bias 203 Participation were selected on a …. rate by the participants) could be considered as a limitation. This could be transferred to the Discussion. However, the authors need to expand on the employed probabilistic sampling

**Response:** thank you for highlighting this to us. We had in the previous version of this manuscript included this paragraph under study limitation in the discussion section but was advised by one of the reviewers to move it to the methodology section. We have now moved it back to the discussion section under study limitation and it now reads.

“Participation was on a voluntary basis therefore the survey had potential for self-selection bias by community members who are particularly concerned about the pandemic. However, probabilistic sampling (stratified Sampling method) was employed. The study also used self-administered questionnaires and the disadvantages of self-administered questionnaires is low response rates, exclusion of those who cannot read and write, and that the researcher cannot couch for the validity of the responses from self-administered surveys. However in our study the response rate was high more than 95 % and also around 95.7% of participants had at least attended primary school level.”

**Comment 4**: The strength of this study is the fact that the authors developed the study questionnaire and it dispensed in both languages (English and Setswana). It would be nice if the authors insert one paragraph or so on the development and validation of the questionnaire. How many items were on the questionnaire? Maybe identify the themes covered (e.g., Demographic information, Cognitive Factors, Experiential Factors, and Socio-cultural factors). The authors could give examples or rationale for each factor.

**Response:** Thank you for highlighting this to us. We have include a paragraph as advised by the reviewer and the paragraph reads:

“To ascertain quality, the questionnaire was pretested before the final draft was made. The development of the questions was guided by the WHO Technical Advisory Group on Behavioural Insights and Sciences for Health paper entitled “Behavioural Considerations for Acceptance and Uptake of COVID-19 Vaccines”. The draft contained about 7 themes and some were dropped because some of the questions were similar or a repetition of questions in other themes. The final version contained Demographic information, Cognitive Factors, Experiential Factors, and Socio-cultural factors. The final version was translated into the native official language and back into English language.”

**Comment 5:** One item of the questionnaire (Section B – Cognitive Factors) B1 asks, “Are you suffering from any of the following ….). I am not sure whether this cognitive factor or is it?

**Response:** Thank you for this clarification. The question is not part of cognitive factors. It is an error that in the questionnaire it was under cognitive factors. The question asks about Comorbid factors of the participants. During the analysis this error was noted and it was not analysed or interpreted as a cognitive factor.

**Comment 6:** I would beg to differ here from other reviewers by recommending the deletion of Figure 1 and Figure 1. First of all, this manuscript is already a bulk one. Also, these figures do not add anything new. This information could be simply narrated in the text.

**Response:** Thank you for this clarification. I have removed figure 1 from the manuscript and replaced it with a text (Narration of the results) as advised by the reviewer. Note that we had in our original manuscript narrated this section and was advised by other reviewers to insert a figure. The edited section now reads

“Three thousand, six hundred and eighty nine (3689) out of five thousand and twenty seven (5027) Participants Figure 1shows that out of 5 027 participants, 3689 were willing to take the vaccine resulting in the acceptance rate of the COVID-19 vaccine in Botswana of 73.4% (CI: 72.2%, 74.6%)”

Overall, this is important and well conducted work and I would advocate publication if those minor issues can be properly addressed.

**Reviewer #2:**

This is surely an improved version of the manuscript. These few things I believe if addressed will make it even better:

**Comment 1**. Generally your table titles need to be improved such that the tables can stand alone and still be readily meaningful to the reader e.g. Tables 1 and 3 could end with (N= 5300). Table 4 title is difficult to understand so rephrase it to be complete and clearly understood. Most titles currently end with “in Botswana” but this was just among the study participants so commonly that is what the title should indicate, something like ‘among study participants in Botswana’

**Response:** Thank you for this clarification. We have corrected all the tables as advised by the reviewer. The new table titles are as follows

“

- Table 2: Socio-Demographic characteristics of participants (n=5300)
- Table 3: Factors associated with COVID-19 vaccine acceptance rate and safety perception among participants in Botswana. (n=5300)
- Table 4: Willingness to receive COVID-19 vaccine against cultural and religious believes among participants in Botswana
- Table 5: The preferred and trusted source of information dissemination among participants in Botswana
- Table 67: Vaccine acceptance rate and safety ofby COVID-19 vaccine by districts in Botswana.”

**Comment 2**. Even though you agreed to attach the questionnaire as supplementary file, there is still the need in the methods section to briefly describe the development of the questionnaire, the content and any piloting processes etc.

**Response:** Thank you for highlighting this to us. We have include a paragraph as advised by the reviewer and the paragraph reads:

“To ascertain quality, the questionnaire was pretested before the final draft was made. The development of the questions was guided by the WHO Technical Advisory Group on Behavioural Insights and Sciences for Health paper entitled “Behavioural Considerations for Acceptance and Uptake of COVID-19 Vaccines”. The draft contained about 7 themes and some were dropped because some of the questions were similar or a repetition of questions in other themes. The final version contained Demographic information, Cognitive Factors, Experiential Factors, and Socio-cultural factors. The final version was translated into the native official language and back into English language.”

3. You have modified to say, this was self-administered questionnaire, does that imply that everyone who participated could read and write in English or the local language used? That would mean that anyone who could not read and write the 2 languages was excluded? You still need to clarify this process.

Response: Thank you highlighting this to us. We have included this in our limitation to read

“The study also used self-administered questionnaires and the disadvantages of self-administered questionnaires is low response rates, exclusion of those who cannot read and write and that the researcher cannot couch for the validity of the responses from self-administered surveys. However in our study the response rate was high more than 95 % and also around 95.7% of participants had at least attended primary school level”

**Comment 4.** Tables 5 and 6 can easily be one table

Response: Thank you for highlighting this to us. We have combined table 5 and 6 and also the narratives of the 2 tables. The new edited section reads as follows:

e. “**Preferred and trusted mode of COVID-19 vaccine related communication**

Table 5 shows that radio ranked as the number 1 (50.3%) preferred source of information followed by television. The table also shows that the majority (53.3%) of the participants trusted the government most as the source of information for COVID-19, followed by World Health Organization (WHO).

**Table 5: The preferred and trusted source of information dissemination among participants in Botswana**

|  | **Preferred source of information dissemination** | | **Most trusted source of information** | | |
| --- | --- | --- | --- | --- | --- |
| **Source of information** | **Frequency (%)** | **Rank** | **Source of Information** | **Frequency** | **Rank** |
| Radio | 2664 (50.3) | 1 | Government | 2826 (53.3) | 1 |
| Television | 1412 (26.6) | 2 | WHO | 1892 (35.7) | 2 |
| Social media | 932 (17.6) | 3 | Social media | 1050 (19.8) | 3 |
| Newspaper | 470 (8.9) | 4 | Internet | 435 (8.2) | 4 |
| Internet | 453 (8.5) | 5 |  |  |  |

“

5. Now you have multivariate analysis results so I would expect that to influence what is presented in the abstract results section but I don’t seem to see that.

Response: Thank you for this clarification. We have re-edited our abstract and the new edited abstract reads:

“**Background**: The COVID-19 disease burden continues to be high worldwide and vaccines continue to be developed to help combat the pandemic. Acceptance and risk perception for COVID-19 vaccines is unknown in Botswana despite the government’s decision to roll out the vaccine nationally.

**Objectives**: This study aims to assess the acceptance rate and risk perception of COVID-19 vaccines amongst the general population in Botswana.

**Methods:** We interviewed 5300 adults in Botswana from 1-28 February 2021 using self-administered questionnaires. The main outcomes of the study were vaccine acceptance and hesitancy rates. Demographic, experiential and socio-cultural factors were explored for their association with outcome variables.

**Results:** Two-thirds of the participants were females (3199), with those aged 24-54 making the highest proportion (61%). The acceptance rate of COVID-19 vaccine was 73.4% (95% CI: 72.2%-74.6%) with vaccine hesitancy at 31.3% (95% CI: 30.0%-32.6%). When the dependent variable was vaccine acceptance, males had higher odds of accepting the vaccine compared to females (OR=1.2, 95% CI: 1.0, 1.4). Individuals aged 55-64 had high odds of accepting the vaccine compared to those aged 65 and above (OR=1.2, 95% CI: 0.6, 2.5). The odds of accepting the vaccine for someone with primary school education were about 2.5 times that of an individual with post graduate level of education. Finally, individuals with comorbidities had higher odds (OR=1.2, 95% CI: 1.0, 1.5) of accepting the vaccine compared to those without any underlying conditions. **Conclusion**: This study demonstrated a high acceptance rate for the COVID-19 vaccine and a low risk perception in Botswana. In order to achieve a high vaccine coverage and ensure a successful vaccination process, there is need to target populations with high vaccine hesitancy rates. A qualitative study to assess the factors associated with vaccine acceptance and hesitancy is recommended to provide an in-depth analysis of the findings.

Word Count: 299 (Target 300).”

All authors have seen and approved the manuscript, and have contributed to the work. There are no copyright restrictions. No authors have conflicts of interest to disclose.

Thank you for considering our revised manuscript. Please contact me for additional information.

Sincerely,

Lebapotswe B Tlale

Public Health Specialist

Ministry of health and Wellness

Cell: +267 71416607 and +267 73340732

Email: cypro330@yahoo.com
