# Supplementary material for: Acceptance rate and risk perception towards the COVID-19 vaccine in Botswana
Source: PLoS One. 2022 Feb 4;17(2):e0263375. doi: 10.1371/journal.pone.0263375 (PMC8815939; doi:10.1371/journal.pone.0263375)
Supplement: Attachment — Submitted filename: Response to reviewers.docx [file pone.0263375.s006.docx]

Dr Lebapotswe Bahumi Tlale, MBBS

4^th^ October 2021

David Teye Doku
Academic Editor
PLOS ONE

Dear Sir

**RE: Manuscript ID PONE-D-21-09673 Acceptance Rate and Risk Perception towards the COVID-19 Vaccine in Botswana: A Cross-Sectional Study. PLOS ONE**

Thank you for your and the reviewer’s thoughtful review of our manuscript. With these in mind we are pleased to submit a revised manuscript for publication in the PLOS ONE Journal. The manuscript has not been published, and is not being considered for publication elsewhere.

These are our responses to each point raised by the academic editor and the reviewers:

**Editor:**

**Comment 1:** In your response to Reviwer 2, you indicated that 95.7% of your respondents at least attended primary school. Could you explain how the 4.3% of the respondents who did not attend at least primary school responded to your self-administered questionnaire of that nature.

Please clarify this before final decision is reached on your manuscript.

“The study also used self-administered questionnaires and the disadvantages of self-administered questionnaires is low response rates, exclusion of those who cannot read and write and that the researcher cannot couch for the validity of the responses from self-administered surveys. However in our study the response rate was high more than 95 % and also around 95.7% of participants had at least attended primary school level”

**Response:** Thank you highlighting this to us. We have reviewed this section and added the how the 4.3% percent responded and is the new write up is as follows:

“The study also used self-administered questionnaires and the disadvantages of self-administered questionnaires is low response rates, exclusion of those who cannot read and write, and that the researcher cannot couch for the validity of the responses from self-administered surveys. However in our study the response rate was high more than 95 % and also around 95.7% of participants had at least attended primary school level. Four point three (4.3%) percent of the participants did not respond the question on educational background.”

All authors have seen and approved the manuscript, and have contributed to the work. There are no copyright restrictions. No authors have conflicts of interest to disclose.

Thank you for considering our revised manuscript. Please contact me for additional information.

Sincerely,

Lebapotswe B Tlale

Public Health Specialist

Ministry of health and Wellness

Cell: +267 71416607 and +267 73340732

Email: cypro330@yahoo.com
